# Evenness-Richness Scatter Plots: a Visual and Insightful Representation of Shannon Entropy Measurements for Ecological Community Analysis

**DOI:** 10.1128/mSphere.01019-20

**Published:** 2021-04-07

**Authors:** Jeff Gauthier, Nicolas Derome

**Affiliations:** a Institut de biologie intégrative et des systèmes, Université Laval, Québec, Canada; b Département de biologie, Université Laval, Québec, Canada; University of California, Davis

**Keywords:** biostatistics, microbial communities, microbial ecology

## Abstract

Shannon’s entropy is a popular alpha diversity metric because it estimates both richness and evenness in a single equation. However, since its value is dependent on both those parameters, there is theoretically an infinite number of richness/evenness value combinations translating into the same index score. By decoupling both components measured by Shannon’s entropy, two communities having identical indices can be differentiated by mapping richness and evenness coordinates on a scatter plot. In such graphs, confidence ellipses would allow testing significant differences between groups of samples. Multivariate statistical tests such as permutational multivariate analysis of variance (PERMANOVA) can be performed on distance matrices calculated from richness and evenness coordinates and detect statistically significant differences that would have remained unforeseen otherwise. Therefore, plotting richness and evenness on two-dimensional (2D) graphs gives a more thorough understanding of how alpha diversity differs between groups of samples.

## OPINION/HYPOTHESIS

Quantifying species diversity is a fundamental theme of ecology. Although there are several definitions of it (alpha, beta, and gamma diversity), it is most often described in terms of alpha diversity, e.g., richness (the number of species) and evenness (a measure of how the species’ relative abundances tend to be uniformly distributed) within a community or habitat (1).

To summarize and compare the alpha diversities of two ecological communities, researchers frequently use scalar diversity indices. As they reduce the dimensionality of complex multivariate data into a scalar number, diversity indices can be compared using null hypothesis tests or confidence intervals (2). However, there is a myriad of those indices, each measuring different parameters, making the direct comparison of values from different indices difficult or even impossible. Some strictly measure species richness such as observed richness, Chao1, and ACE estimators (3) while others estimate alpha diversity as a phylogenetic metric (e.g., Faith’s phylogenetic diversity [PD] index). Other metrics, such as Shannon’s entropy (*H*′), englobe richness and evenness into a single metric. This index is unarguably one of the most popular metrics in community ecology, alongside Simpson’s diversity index (λ), even though there are yet no clear guidelines on which diversity index should be used (4).

Shannon’s entropy (*H*′) is defined as follows:

(1) $$H^{\text{′}}=-\overset{S}{\underset{i=1}{\sum }}p_{i}\text{ln}p_{i}$$

where *S* is the total amount of species in a biome and *p_i_* is the relative abundance (proportion) of species *i*. However, since it measures both richness and evenness in a single equation, there is theoretically an infinite number of richness/evenness value combinations translating into the same index score. Furthermore, richness and evenness may covariate positively, but also negatively. For example, in a 2020 study on microbiota dynamics in yellow perch (Perca flavescens) exposed to trace cadmium contamination, “decreasing richness and increasing evenness were observed” (5). There is no way of detecting whether evenness/richness covariance is either positive or negative by using only Shannon’s entropy, because it outputs the level of uncertainty in the species profile of a community, not how many species there are or how even their distributions are (6). To do so, Shannon’s entropy is usually compared alongside one or more indices that measure either richness or evenness (7, 8).

Here is a fictitious example with two simple mock communities to illustrate this issue (Table 1). Both have near-identical Shannon indices despite a very different community composition (1.609 and 1.608, respectively). This is because Shannon’s entropy binds richness ($\Sigma_{i=1}^{K}\;i$) and evenness (−*p_i_*ln*p_i_*) together in a single equation. Decoupling its components would yield a more detailed overview of alpha diversity than what Shannon’s entropy would reveal alone. This can be achieved by visualizing richness and evenness on two-dimensional (2D) graphs, where each parameter would be assigned to an orthogonal axis. Such graphs would also eliminate the need for comparing richness and evenness indices side by side, as the two concepts are visualized simultaneously and derived from the formula for Shannon’s entropy.

**TABLE 1:** Relative species proportions in communities 1 and 2

Species	Proportion in community 1	Proportion in community 2
Species 1	0.2	0.500
Species 2	0.2	0.100
Species 3	0.2	0.100
Species 4	0.2	0.100
Species 5	0.2	0.095
Species 6		0.050
Species 7		0.030
Species 8		0.025

## METHODOLOGY

### Deriving species richness.

The simplest definition of species richness is the total amount of species found in a community (the term *S* in equation 1). Although several definitions of species richness have been formulated (e.g., Chao1, ACE, etc.), for the sake of simplicity, we will illustrate species richness here by its simplest definition (i.e., the number of observed taxa without extrapolating rare taxa).

### Deriving evenness.

Deriving evenness from Shannon’s entropy is not as obvious as deriving richness. The “evenness” component would be calculated using relative abundances (*p_i_*). The ln-transformation of *p_i_* in Shannon’s entropy’s formula narrows the range (and therefore the impact) of extreme values and still weighs high relative abundances as “high” and low abundances as “low.”

A way to estimate evenness would be to calculate the median of summation operand in Shannon’s entropy formula (−*p_i_*ln*p_i_*). The median is an efficient trend indicator that is not affected by outlier values as the arithmetic average is (9). Furthermore, a very uneven community would be expected to have a very low median −*p_i_*ln*p_i_*, whereas a perfectly even community would have a median −*p_i_*ln*p_i_* equal to any −*p_i_*ln*p_i_*.

As expected, in Table 1, evenness in community 1 (0.321…) is higher than in community 2 (0.227…). However, this formulation is not fully satisfying, as a perfectly even community (community 1) should have an evenness index of 1, and a very unequal sample should have an evenness index nearing zero. In our example, indices of even and uneven communities, although different, are very close. This issue could be solved by normalizing the median −*p_i_*ln*p_i_* by the highest −*p_i_*ln*p_i_* value. Normalized values of evenness become 1.000 for the perfectly even community 1 and 0.655 for the uneven community 2. To further validate this index, here called “normalized-median evenness,” let two additional communities be created, which are even more extreme than the first two, but each having three species in order to keep richness constant (Table 2). Normalized-median evenness clearly differentiates communities 2 and 4, both of which are very different in terms of richness and evenness. Here is its definition:

(2) $$\text{NME}=\frac{\text{median }\left(-p_{i}\text{ln}p_{i}\right)}{\text{max }\left(-p_{i}\text{ln}p_{i}\right)}$$

where NME is normalized-median evenness, *p_i_* is the relative abundance of species *i*, and max is the maximum value of −*p_i_*ln*p_i_*.

**TABLE 2:** Relative species composition of mock communities 1 to 4 along with their product-ln-transformations

Species	Proportion^*a*^ in community 1	Proportion^*a*^ in community 2	Proportion^*a*^ in community 3	Proportion^*a*^ in community 4
Species 1	0.2 (0.322)	0.5 (0.347)	0.333 (0.366)	0.95 (0.049)
Species 2	0.2 (0.322)	0.1 (0.230)	0.333 (0.366)	0.095 (0.224)
Species 3	0.2 (0.322)	0.1 (0.230)	0.333 (0.366)	0.005 (0.026)
Species 4	0.2 (0.322)	0.1 (0.230)		
Species 5	0.2 (0.322)	0.095 (0.224)		
Species 6		0.05 (0.150)		
Species 7		0.03 (0.105)		
Species 8		0.025 (0.092)		
Richness	5	8	3 (1)	3
Evenness (NME)	1.000	0.655	1.000	0.218
Evenness (Pielou)	1.000	0.773	1.000	0.272

a Relative proportions (*p_i_*) are indicated along with their product-ln-transformations (−*p_i_*ln*p_i_*) in parentheses.

NME is similar in principle to, but different from, Pielou’s evenness (*J*′), a well-known index (10) that expresses the ratio between a community’s *H*′ value and the value *H*′ would take if the community was perfectly even (*H*′_max_). Mathematically:

(3) $$J^{\text{′}}=\;\frac{H\text{′}}{H{\text{′}}_{\text{max}}}$$

where *H*′ is Shannon’s entropy and *H*′_max_ is its maximum possible value (if every species was equally likely). In such a case, *p_i_* equals *1/S* which makes *H*′_max_ equal to:

(4) $$H{\text{′}}_{\text{max}}=-\overset{S}{\underset{i=1}{\sum }}\frac{1}{S}\ln\frac{1}{S}=\ln S$$

where *S* is the raw number of species or richness.

Pielou’s evenness is constrained between 0 and 1. It does not consider actual species proportions from the measured community, instead expressing *H*′ as a ratio of a maximum theoretical value that is never seen in practice.

Unlike Pielou’s index, NME’s numerator and denominator, respectively, represent the median and maximum value of −*p_i_*ln*p_i_* from a given biome. Furthermore, NME’s calculation is independent from the calculation of Shannon’s entropy, i.e., one does not have to calculate *H*′ to calculate NME.

Using the fictitious community examples shown above, NME better separates the uneven and very uneven communities (biomes 2 and 4), whereas the perfectly even communities (biomes 1 and 3) have evenness values equal to 1 using whichever index (Table 2).

### Graphical representation.

**FIG 1:**
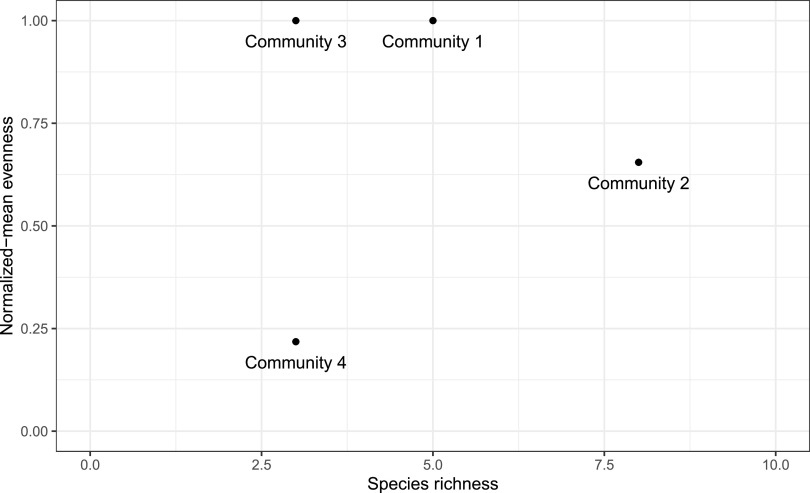
Normalized-Median Evenness versus Richness plot for mock communities 1 to 4.

By having both components of Shannon’s entropy untangled, two samples can be compared simultaneously, even if they possess identical Shannon indices. This can be achieved by plotting richness and normalized-median evenness on a scatter plot, where each metric would correspond to a different axis (Fig. 1). Communities 1 and 2 can be fully differentiated, even though they have the same Shannon index but different richness and evenness terms. Communities 3 (very even) and 4 (very uneven) are also fully differentiated from each other, even though their species richness is the same.

### Example with a larger mock data set.

**FIG 2:**
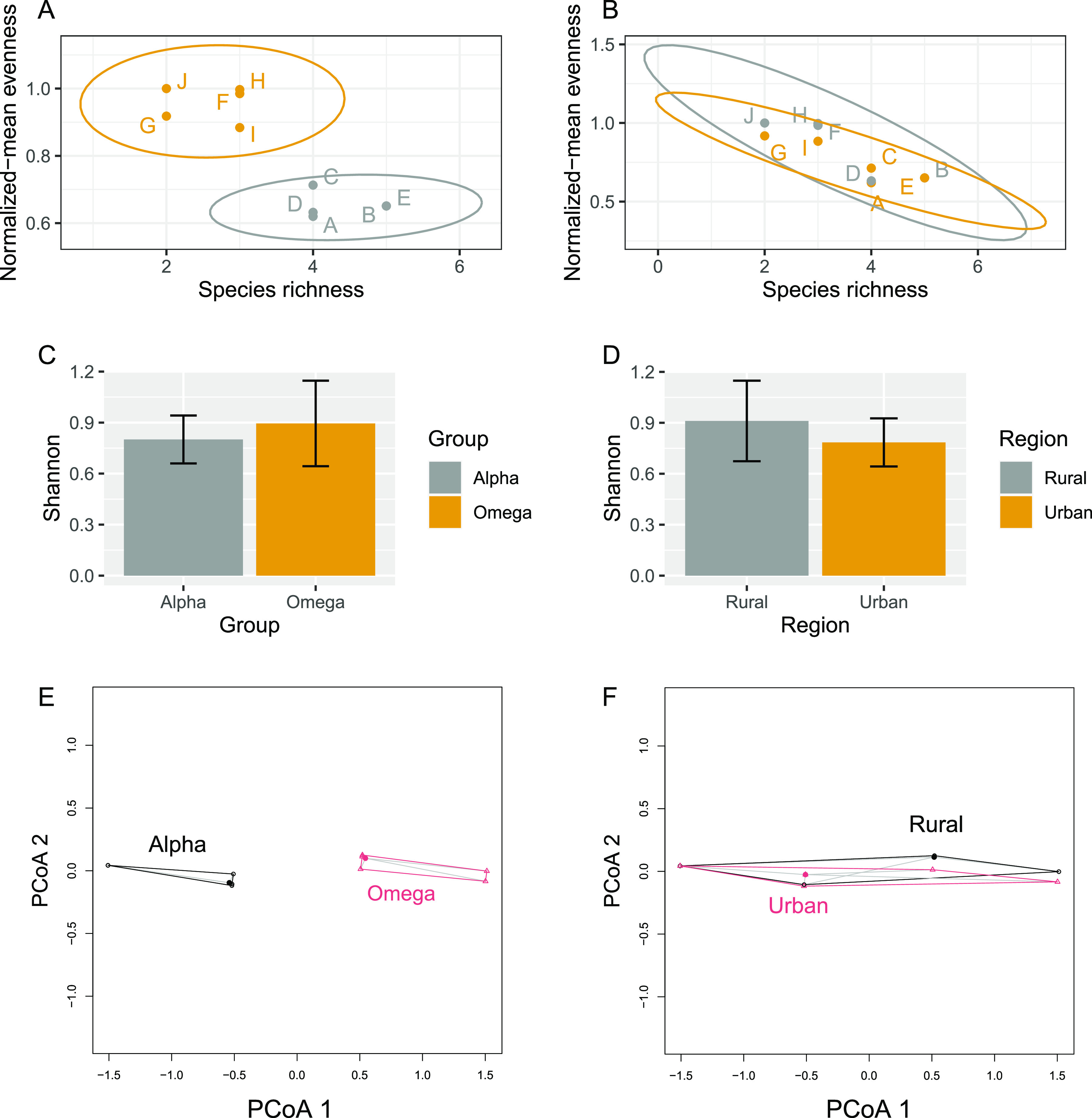
Alpha diversity for fictional communities A to J. (A and B) Normalized-Median Evenness versus Richness plot for grouping factors Group and Region. Ellipses = 95% confidence intervals. (C and D) Mean Shannon index + 95% CI for grouping factors Group and Region. (E) Multivariate dispersion plot of the evenness-richness coordinates with respect to Group. (F) Multivariate dispersion plot of the evenness-richness coordinates with respect to Region.

Let there be a mock data set where the diversity of two groups (Alpha and Omega) of five samples (A to J) belonging to two regions (Urban, Rural) is compared (Table 3). All previously discussed diversity indices, or their components, have been precomputed. Now let the data be plotted on a Richness versus Normalized-Median Evenness plot as previously described, with 95% confidence ellipses for each group (Fig. 2A). A clear separation between samples from the Alpha and Omega groups can be made, which would not have been possible by comparing their Shannon indices alone (Fig. 2C). Note how means and confidence intervals (CIs) overlap. Confidence ellipses may be used to detect statistically significant differences between groups, but they are not very useful for assessing the effect of several grouping variables at the same time. To illustrate this factor, let another grouping factor (Region) be included in fictional samples A to J mentioned above (Table 3; Fig. 2B and D).

**TABLE 3:** Species composition and various diversity metrics of a larger fictional data set of 10 biomes belonging to two groups (Alpha and Omega) and two regions (Urban, Rural)

Biome	Group	Region	p_1_	p_2_	p_3_	p_4_	p_5_	Sum(*p_i_*)	Richness	Evenness (NME)	Shannon
A	Alpha	Urban	0.7	0.2	0.05	0.05		1.00	4	0.620	0.871
B	Alpha	Rural	0.8	0.1	0.05	0.0045	0.005	1.00	5	0.651	0.725
C	Alpha	Urban	0.8	0.1	0.05	0.05		1.00	4	0.713	0.708
D	Alpha	Rural	0.6	0.3	0.05	0.05		1.00	4	0.632	0.967
E	Alpha	Urban	0.8	0.1	0.05	0.04	0.01	1.00	5	0.651	0.733
F	Omega	Rural	0.4	0.3	0.3			1.00	3	0.985	1.089
G	Omega	Urban	0.6	0.4				1.00	2	0.918	0.673
H	Omega	Rural	0.4	0.35	0.25			1.00	3	0.997	1.081
I	Omega	Urban	0.6	0.25	0.15			1.00	3	0.884	0.938
J	Omega	Rural	0.5	0.5				1.00	2	1.000	0.693

It is possible to use the evenness and richness values to compute a Euclidean distance matrix, which can be used as input for permutational multivariate analysis of variance (PERMANOVA) (11). A PERMANOVA was computed (99 permutations total) with the adonis() function from the vegan package (12) using Richness and Evenness values as response variables and Group and Region as explanatory variables. It revealed a significant effect of Group on alpha diversity (*F* = 14.3, *R*^2^ = 0.70, *P* = 0.02), but no significant Region effect (*F* = 0.32, *R*^2^ = 0.02, *P* = 0.59) and no significant interaction between Group and Region (*F* = 0.04, *R*^2^ = 0.0002, *P* = 0.88). If a conventional two-factor ANOVA had been performed using Shannon’s entropy as a response variable, no significant effect on alpha diversity would have been detected, regardless of the grouping factor (0.2 < *P* < 0.92). Multivariate dispersion plots (Fig. 2E and F) show that richness-evenness coordinates have similar dispersions, indicating constancy of variance and the respect of PERMANOVA’s assumption of homoscedasticity, even though this multivariate test is robust to nonconstancy of variance in balanced designs (13).

The next section describes an example of real data analysis using Evenness-Richness scatter plots and related statistical analyses. Briefly, the use of those graphs allowed a more thorough view of alpha diversity than using Shannon’s entropy. Moreover, we identified a clustering effect caused by the pooling of data obtained through multiple next-generation sequencing technologies. This effect was shown to be significant with PERMANOVA.

### Analysis of a real data set: enterotypes of the human gut microbiome (2011). (i) Introduction to the data set.

Published in *Nature* in 2011, the work of Arumugam et al. compared the fecal microbiota from 22 subjects using complete shotgun DNA sequencing (14). The authors further compared these microbial communities with the fecal communities of subjects from other studies. A total of 280 fecal samples/subjects are represented in this data set, and 553 microbial genera were detected. The authors claim that the data naturally clump into three community-level clusters, or “enterotypes,” that are not immediately explained by sequencing technology or demographic features of the subjects. These data are included into the R package phyloseq (15) as an example data set.

**FIG 3:**
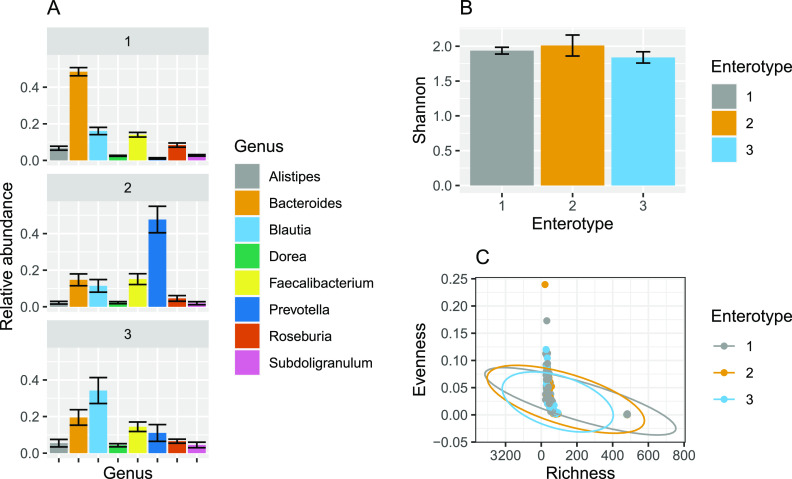
Impact of enterotype on alpha diversity within the human gut microbiota. (A) Relative proportions of the top 8 most abundant genera across the enterotype data set. Error bars indicate 95% confidence intervals. (B) Mean Shannon index of enterotype samples grouped by enterotype. Error bars indicate 95% confidence intervals. (C) Evenness-Richness plot of enterotype samples grouped by enterotype. Ellipses = 95% confidence intervals.

When studying the top 10 most abundant genera across the enterotype data set, we see that each enterotype is dominated by distinct subsets of genera (Fig. 3A). Enterotype 1 is dominated by *Bacteroides* spp., whereas enterotypes 2 and 3 are dominated by *Prevotella* spp. and *Blautia* spp., respectively. Despite the very different top genus abundance profiles, the three enterotypes appear very similar in terms of alpha diversity when measured with Shannon’s entropy (Fig. 3B).

### (ii) Evenness-Richness graph analysis.

When alpha diversity across enterotypes is visualized with Evenness-Richness scatter plots instead, we see that the confidence ellipses of each enterotype group are entirely overlapping (Fig. 3C). However, there are two completely distinct clusters visible on this figure, each being composed of samples from various enterotypes. In the right-side cluster (here named cluster 2), there is a higher proportion of samples from enterotype 1 (Table 4).

**TABLE 4:** Number of samples belonging to enterotypes 1, 2, and 3 in both clusters seen on the previous figure

Cluster	Samples from enterotype 1	Samples from enterotype 2	Samples from enterotype 3	Total
Cluster 1	105	26	55	186
Cluster 2	63	9	13	85
Total	168	35	68	271

**FIG 4:**
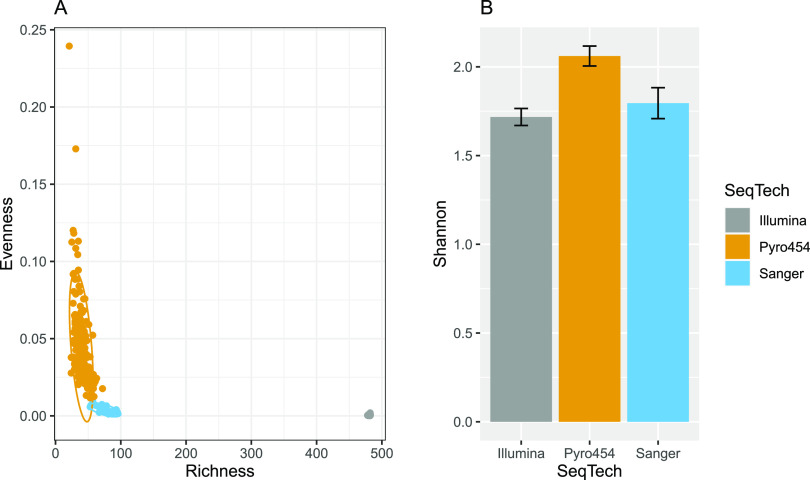
Alpha diversity of enterotype samples grouped by sequencing technology. (A) Evenness-Richness plot. Ellipses indicate a confidence level of 95%. (B) Mean Shannon index of enterotype samples grouped by sequencing technology. Error bars indicate 95% confidence intervals.

There is a significant discrepancy in the relative proportions of enterotypes in the two clusters (χ^2^ = 8.1945, *P* < 0.02). Given that those clusters are differentiated along the Richness axis, there appears to be a systematic bias on the assessment of richness between the two clusters. Interestingly, the enterotype data set includes data obtained through three different sequencing technologies (i.e., Sanger, 454, and Illumina). An Evenness versus Richness plot with samples labeled by sequencing technology revealed that cluster 2 is made of all the Illumina samples of the data set (Fig. 4A).

Richness in Illumina samples is about 1 order of magnitude higher than in Sanger samples. This may be reflective of the high throughput of Illumina sequencing relative to Sanger sequencing (16). In contrast, evenness appears lower in Illumina samples than in Sanger and 454 samples. Those differences in sequencing technologies could not have been detected while using Shannon’s entropy alone.

**FIG 5:**
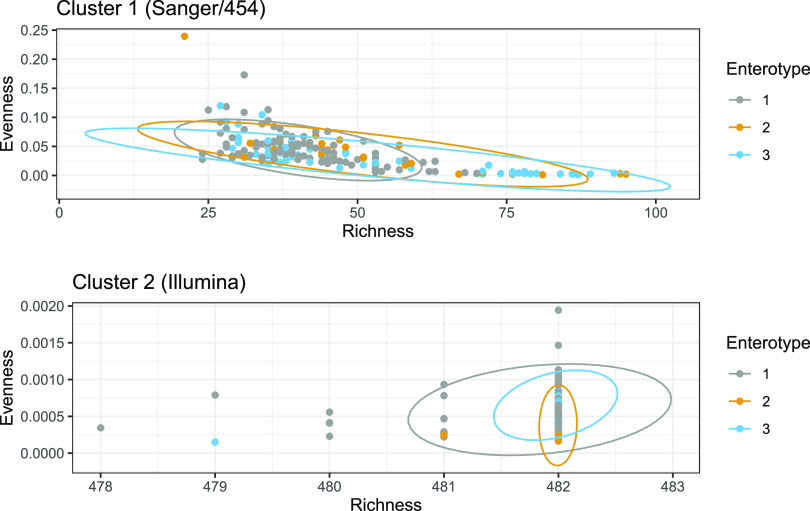
Evenness-Richness plot of samples in cluster 1 (Sanger/454) and cluster 2 (Illumina).

The average Shannon index of 454 samples is significantly different from the two other groups, whose means and CIs completely overlap (Fig. 4B), despite that Illumina and Sanger samples differ in orders of magnitude in evenness. If the data from cluster 1 (Sanger/454 samples) and cluster 2 (Illumina samples) were analyzed separately, the conclusions made on the enterotypes’ alpha diversity would have been different (Fig. 5).

**FIG 6:**
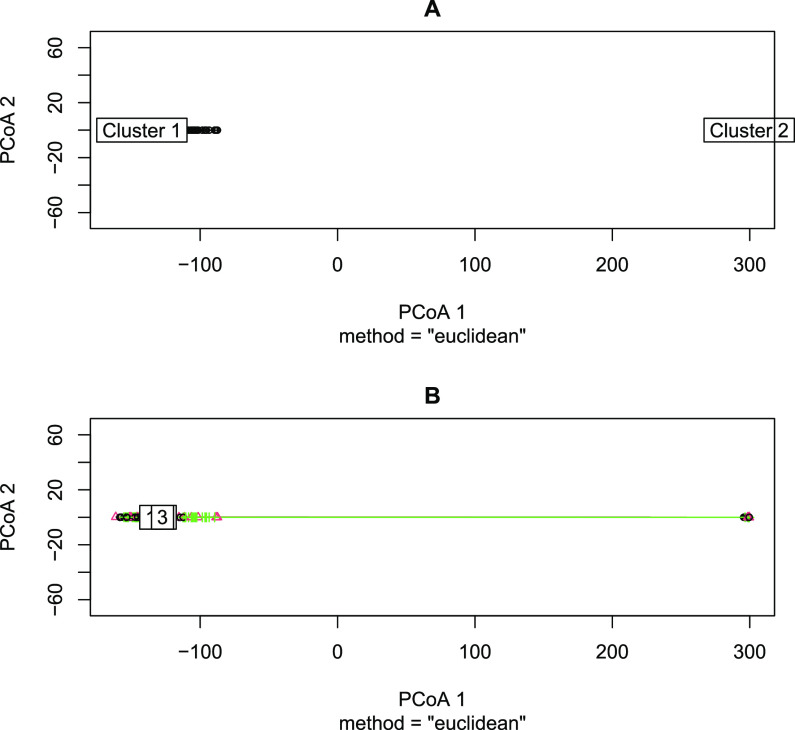
Multivariate dispersion plot of the evenness-richness coordinates with respect to cluster (A) and enterotype (B).

The clustering effect caused by sequencing technology and its influence on the distribution of enterotypes were further assessed with a PERMANOVA. A total of 99 permutations were calculated, with the Evenness-Richness Euclidean distance matrix as a response object and Cluster as explanatory variable. There is a strong and significant effect of Cluster (*R*^2^ = 0.99, *P* < 0.01). The Enterotype variable could not be used for PERMANOVA because of nonconstancy of variance (Fig. 6). Using an Evenness versus Richness plot to visualize alpha diversity allowed us not only to detect but also to quantify this clustering effect caused by sequencing technology in the enterotype data set.

### Advantages and limitations. (i) Use of richness as a component.

The two-dimensional representation of Shannon’s entropy described here uses richness as one of its components. Richness has been shown to be an unreliable alpha diversity metric compared across studies, in part because of the plethora of factors influencing its value, e.g., sampling design, measurement method, sequencing throughput, etc. (17). Here, the choice of species richness as a component was to simplify the presentation of the method. An interesting alternative would be the use of Hill numbers (2), also called “effective number of species.” For example, the first-order Hill number (^1^D) is mathematically related to Shannon’s entropy, is less sensitive to sampling and/or throughput biases, and could provide a less biased alternative to absolute species richness (6).

### (ii) Relationship with other diversity plotting methods.

We are aware that communities can be differentiated by plotting an ordination from a beta diversity distance matrix or by comparing abundances. However, beta diversity does not measure entropy but rather the distance (or turnover) between two community compositions. It is a different concept from alpha diversity (1). Furthermore, beta diversity is usually plotted in ordinations (e.g., principal-component analysis [PCA] or nonmetric multidimensional scaling [NMDS]) whose axes are not directly interpretable except for how they explain variance. The axes in evenness-richness scatter plots are not ordination components but rather alpha diversity metrics (richness and evenness) which make it possible to visually explain the distance between two data points in terms of richness and/or evenness or both (i.e., alpha diversity increases diagonally toward the top right corner of plots).

### Conclusion.

By comparing Shannon’s entropy alone, groups of samples may be entirely indistinguishable from one another. Moreover, one may overlook methodological biases that may affect the interpretation of alpha diversity analysis, e.g., combining data sets obtained through different sequencing technologies as seen in the enterotype data set. Therefore, plotting the two components that it measures (richness and evenness) on 2D graphs gives a more thorough understanding of how alpha diversity differs between groups of samples. The data can be visualized in a 2D scatter plot where tight grouping indicates similarity between samples. Statistical methods, such as confidence ellipses or PERMANOVA, can be used to detect significant differences between groups, even if their Shannon index is the same.

## MATERIALS AND METHODS

All statistical analyses were performed in RStudio using R v3.4.2. Briefly, mock data sets were prepared using predetermined taxon abundance values in order to best illustrate cases where Shannon indices are identical between two communities despite different richness and evenness values. The enterotype data set was imported from (and analyzed with) the phyloseq package suite for microbiome data analysis (15). Top taxon abundance graphs were generated with a customized version of phyloseq’s plot_bar function which builds mean taxon abundance plots with error bars. Shannon’s entropy and Evenness-Richness scatter plots were generated with package ggplot2 (https://ggplot2.tidyverse.org/) from summarized data remodeled from phyloseq-class objects with the summarySE function from package Rmisc (https://www.rdocumentation.org/packages/Rmisc/). For univariate plots, 95% confidence intervals were calculated either from the summarySE() function or from the ci() function from package gmodels (https://cran.r-project.org/web/packages/gmodels/), while 95% confidence ellipses were calculated within ggplot2 for 2D graphs using the stat_ellipse() function. PERMANOVAs were computed with the adonis() function from the R vegan package (12). Prior to PERMANOVA, the homoscedasticity of Euclidean distances between richness-evenness coordinate pairs was verified using vegan’s betadisper() function for each grouping factor, and then visualized as principal-coordinate analysis (PCoA) plots using R’s default plot() method.

### Data availability.

A full R Markdown version of this article’s source code is available on GitHub: http://www.github.com/jeffgauthier/alpha-diversity-graphs/.
